# The application of evolutionary medicine principles for sustainable malaria control: a scoping study

**DOI:** 10.1186/s12936-016-1446-8

**Published:** 2016-07-22

**Authors:** Denise Ocampo, Mark Booth

**Affiliations:** Department of Anthropology, Durham University, Stockton Rd, Durham, UK; School of Medicine, Pharmacy and Health, Durham University, University Boulevard, Thornaby, UK

**Keywords:** Malaria, Evolution, Control programmes

## Abstract

**Background:**

Current interventions against malaria have significantly reduced the number of people infected and the number of deaths. Concerns about emerging resistance of both mosquitoes and parasites to intervention have been raised, and questions remain about how best to generate wider knowledge of the underlying evolutionary processes. The pedagogical and research principles of evolutionary medicine may provide an answer to this problem.

**Methods:**

Eight programme managers and five academic researchers were interviewed by telephone or videoconference to elicit their first-hand views and experiences of malaria control given that evolution is a constant threat to sustainable control. Interviewees were asked about their views on the relationship between practit groups and academics and for their thoughts on whether or not evolutionary medicine may provide a solution to reported tensions.

**Results:**

There was broad agreement that evolution of both parasites and vectors presents an obstacle to sustainable control. It was also widely agreed that through more efficient monitoring, evolution could be widely monitored. Interviewees also expressed the view that even well planned interventions may fail if the evolutionary biology of the disease is not considered, potentially making current tools redundant.

**Conclusions:**

This scoping study suggests that it is important to make research, including evolutionary principles, available and easily applicable for programme managers and key decision-makers, including donors and politicians. The main conclusion is that sharing knowledge through the educational and research processes embedded within evolutionary medicine has potential to relieve tensions and facilitate sustainable control of malaria and other parasitic infections.

**Electronic supplementary material:**

The online version of this article (doi:10.1186/s12936-016-1446-8) contains supplementary material, which is available to authorized users.

## Background

Since 2000 there has been a substantial increase in global funding and international efforts to combat malaria [1, 2]. As a result, the latest World Health Organization (WHO) reports show a steady decline of malaria incidences and deaths [1, 2]. This success has been achieved by shifting focus from eradication to control [1, 2]. Eradication is still considered possible, through universal and sustainable coverage of drugs, transmission-reducing tools and through strengthening health systems [1].

Despite this optimism, an important question remains as to the sustainability of these interventions to the point of eradication—given that both vectors and parasites are evolving faster than counteractions [3]. Human behaviour is imposing selective pressure on the vector and the pathogen via different pathways [3, 4]. Additionally, co-evolution between parasites, vectors and hosts may have direct consequences on virulence and transmission [3, 5–10].

Resistance to drugs is an outcome of evolutionary processes; consequently, the selection pressures associated with treatment need to be considered within malaria programmes [11]. Studying how parasites, vectors and hosts co-evolve and, by considering what their most probable next developmental phase will be, could allow improved protection and thus an advantage in the battle against malaria [5, 6, 12, 13]. Therefore, the question posed was: are principles derived from evolutionary medicine (EM) [14] being considered in the fight against malaria and used to make interventions more sustainable? Additionally, it was questioned whether there is sufficient collaboration between academic research, programme management and key decision-makers to facilitate sharing knowledge and generate common understanding.

This scoping research project was intended to understand how EM might act as a bridging domain of enquiry amongst stakeholders from research and control programme backgrounds. The results are intended to act as a catalyst and framework for further discussions towards sustainable control.

## Methods

### Research setting and sample

Qualitative interviews were conducted in the fields of malaria research and control. Actors in universities, disease prevention institutions and health partnerships were identified and contacted via email with an explanation of the research and a request for an interview. Everyone who showed willingness was accepted as an interview participant; thereby forming a convenience sample. The interviewees consisted of thirteen people, eight of whom worked in applied malaria programmes and five in malaria research. All participants were stakeholders in the research and control of malaria affecting people living in Africa. The principles of EM were outlined to each individual, when necessary, prior to the delivery of questionnaires. There were more programme managers in the sample as the main focus of the research was the practical application of evolutionary principles. For the research question, it was primarily important to understand what roles the participants played in malaria control.

### Instruments, data collection and analysis

Each participant was informed about the purpose of the study and asked to give consent to have the interviews audiotaped, transcribed and used for the research. The consent and information form can be found in the Additional file 1. Durham University, as well as each participant, has given consent for this study to be published. Semi-structured and guided video or telephone interviews were held as most participants were not located in the UK. All interviews were audiotaped and transcribed. Original transcripts are available from the author.

As a precis to discussion on the potential contribution of EM to sustainable malaria control, participants were asked to respond to a series of questions corresponding to current control practice. The main topics addressed in the interviews were: perceived reasons for the successes and failures of malaria control programmes; first-hand experiences encountered working in this field; first-hand knowledge of the effects of resistance to drugs and insecticide on programme success; the interviewee’s practical experiences of countering these challenges; and their ideas on possible solutions. The topic of EM was then addressed: by asking each interviewee if they considered its principles were already applied in control programmes and if not should they be incorporated. Interviewees were finally asked for their predictions for the future. The topics were chosen to match the main problems in the malaria literature and were tailored to what the interviewees perceived to be problem areas. The wording, structure, style and main focus were adapted to the individual interviewee, their field of speciality, and the nature of the interview. A copy of the interview questions can be found in Additional file 2.

For the presentation of the results of the interviews statements of the academics and programme managers were compared. Participants were categorized into these two groups as this best shows both ends of the spectrum in the malaria community. It should be noted that some interviewees worked in both sectors, but for the purpose of analysis they were categorized according to their present role.

## Results

From the original questionnaire eight compelling areas of reflection and discussion emerged. These were categorized into the following eight domains:

- Reasons for problems and failures in malaria programmes.
- Reasons for successes of malaria programmes.
- Reflections on the importance of drug and insecticide resistance.
- Knowledge about the underlying causes of drug and insecticide resistance.
- Knowledge about the relationship between pathogen, vector and host and the implications for immunity, transmission and virulence.
- Application of EM principles in malaria programmes.
- The relative merits of action or reaction.
- The role of communication between project managers and academia.

Each of these topics is addressed below with a narrative assessment of the questionnaire responses.

### Problems and failures of malaria programmes

Eight programme managers said insufficient funding was the biggest challenge and four said that management of the programmes was the biggest problem. Four researchers said, that in addition to insufficient funding, insecticide resistance was the main problem. The following five points were voiced by both groups: insufficient funding and human resources; insecticide and drug resistance; malfunctioning public health care systems, poor infrastructure; and low surveillance and data collection for monitoring.

One participant from each group felt that the lack of knowledge, and foresight to resistance, in addition to the absence of ways to apply this knowledge was a reason for failure. Both groups also mentioned resistance to the drug artemisinin, and felt that it was the same programme strategies of vector control that were being applied rigidly to every situation which were causing programmes to fail where these tactics were not appropriate. Four of the programme managers pointed out that the lack of political will in countries endemic to malaria was another major cause for programmes to fail.

A difference that came up between the groups was that, whilst the lack of technical knowledge was said to be a problem by one programme manager, the lack of multiple effective interventions was identified as a problem by two researchers. This indicates that interviewees of both groups put some of the responsibility for the problems with malaria programmes onto the other group.

### Success of malaria programmes

The four points that were perceived to be successes and mentioned by both groups were: the introduction of artemisinin-based combination therapy (ACT) and long-lasting insecticide-treated nets (LLINs); improved treatment; the scaling-up of control tools in countries endemic to malaria; and finally the increased funding received in recent times.

### Drug resistance

Three academics interviewed said that drugs were currently overused and misused. A programme manager as well as an academic emphasized the importance of correct drug usage, the development of new drugs and monitoring, but considered insecticide resistance to be the greater problem. Two programme managers expressed hope that resistance to Artemisinin would not spread to Africa but would remain contained in South East Asia through elimination of the parasite. Three researchers conversely pointed out that resistance to drugs will inevitably occur and measures need to be taken proactively.

### Insecticide resistance

Problems with insecticide resistance arising through the usage of insecticides in public health interventions and agriculture are being observed by both project managers and academics. Both groups agreed that monitoring was necessary in order to stay informed and take appropriate action but there is currently a lack of monitoring. While programme managers put an emphasis on the need for new insecticides they also agreed that alternative control methods are necessary. They saw the intense usage of insecticides in agriculture as a source of resistance. This issue was heightened by the fact that there is very little communication and cooperation between the agriculture and health sectors. As food production has a higher priority than disease management there is currently no plan to change agricultural insecticides.

There was a divide amongst programme managers about the application of insecticides. Two participants of this group said that rotating insecticides was a good technique to avoid resistance while another argued that this more expensive application of insecticides is futile if agriculture continued to use the same active ingredients as public health for insecticides. One of the reasons given for the slow response to resistance was the lack of available insecticides. Pyrethroids were the only class of insecticides recommended by the WHO for bed nets as they were safe, cheap and extremely effective. As a result of their exclusive usage, resistance occurred. One of the researchers said that resistance was the price that was paid for the huge success of achieving high coverage and reducing mortality rates by 47 % globally [2]. However, others fear that this success will be lost if resistance is not taken seriously.

A further reason for the slowness of the reaction to resistance was given by one of the programme managers who explained that the development of insecticide resistance is much more difficult to detect than that of drug resistance. Unlike drugs that go through a standardized process of control phases, insecticide development does not have the same procedure. Scientists perform tests at random on insecticides which then need to be approved by the WHO. This makes the development of insecticides and other control tools unreliable.

### Resistance as a result of evolution

Academics and programme managers agreed that, although resistance was anticipated, it was not planned for and that programmes lacked foresight. This results in programmes having limited choices once resistance occurred. Both groups agreed that resistance occurs as a result of evolutionary processes. They also concurred that this issue, if not addressed, would lead to higher mortality rates. Furthermore, both groups agreed that the actions currently taken were too slow. Resistance itself however, is perceived differently by the two groups. Two programme managers did not think resistance is due to failures of the interventions and programmes did not have many options once resistance did occur. However, a researcher pointed out that resistance was a failure as it was anticipated and counter measures happened too slowly.

### The application of EM principles in malaria control programmes

There was disagreement amongst participants with respect to whether or not evolutionary principles are already being considered within control programmes. Whilst two programme managers said this was the case, others in both groups stated that this was not; the reason given was the general lack of human and financial resources. It was stated by four participants that people working on the front line of malaria control were overburdened with the urgent task of reducing transmission, mortality and morbidity with the tools they had, evolutionary approaches are therefore not given priority. One researcher emphasized that academics understood this problem and he agreed that interventions should be delayed for the sake of research. While two programme managers said it is logistically extremely difficult to conduct studies to make interventions evolution-proof, both sides saw the necessity to integrate EM principles to prolong the life span of control tools.

There were variable answers to the question whether EM principles are considered globally. Evolutionary planning was perceived by some of the interviewees as something to be considered long-term and globally, to make sure that the short-term achievements are not lost. Three participants from both groups said that, especially for drugs, evolutionary principles are taken into account by the WHO and their recommendations are implemented into programmes. While other interviewees in both groups said that they are currently not incorporated because the main priority is getting coverage; only once transmission is reduced, other aspects can be considered.

Academics thought it was necessary for the public to know and understand that malaria control is a process and that there is no one simple lasting solution. However, one programme manager counter argued that it is difficult enough to get people to use control tools and take medicines; evolutionary information would only harm the process of getting quick and rapid coverage. Both academics and programme managers agreed that programmes do not have the resources to look into the future and try to do the best with the tools they have. Nevertheless, both groups agreed that researchers need to make control tools evolutionary-proof prolonging their effectiveness by making it difficult for vectors to develop resistance. A programme manager suggested placing some of the responsibility with researchers to develop new tools such as insecticides, that are not fast acting neurotoxins and do not kill immediately hence not putting a strong selection pressure on the vector.

Overall, it was acknowledged by the majority of members of both groups that poor monitoring, lack of understanding, standard strategies applied continually and in different environments lead to interventions that may be more harmful than helpful by not taking the evolutionary history of the pathogen, vector and host into account. Both groups agreed on the fact that research results have to be made practical and that academics have to make them accessible and applicable for programmes.

One interviewee explained that programmes often do not run long enough to study the long-term effects interventions have on pathogen, vector, and host. Thus, the decision-makers fail to see the contribution EM principles could make. This view can be seen in the statements below:

“*The way our programmes are structured we don’t take evolution into account because we are too short sighted, literally*” (Programme Manager, Telephone interview, May 5, 2015).

“*If I was a programme manager in an African country and I was severely hit by malaria and I had X access to X thousands of dollars I would probably choose to protect my population with whatever tools I have at the moment*” (Programme Manager, Telephone interview, May 6, 2015).

“*If you can reduce transmission by 50* *% or even 30* *% then the tools have a better chance of working to prevent and eliminate malaria*” (Programme Manager, Telephone interview, May 14, 2015).

Although the Multisection Action Framework for Malaria from RBM calls for collaboration from different sectors, results of this study indicate a general disconnect between academics and programme managers when it comes to evolutionary principles. One researcher said that it was important for people to realize that tools do not last forever as parasites and vectors evolve. While in contrast a programme manager pointed out that this kind of message would reduce trust in the control programmes.

### Communication between academia and practical application

The information acquired from the interviews revealed a disconnection between the theoretical measures that would slow down resistance and sustain success, and what can practically be done. The interviews furthermore revealed some of the reasons for this and exposed the lack of cooperation between the different groups. There were disagreements within the groups. Both the programme manager group and academia group included people saying that communication already has been improved and some saying there is still a divide between these two sectors. Academics and programme managers complained about the lack of communication and cooperation between the groups. One of the interviewees shared their impression of an annual RBM meeting, stating that evolutionary presentations were so technical and mathematical that only about 20 % understood what was being said. Therefore, the information given was lost.

One of the researchers was frustrated that research results with possible important implications for malaria control was published in papers but not extended to the people who need to know. This participant argued that researchers need to take responsibility for communicating results in a simple and accessible manner. This opinion and the fact that the process of putting research into practice is currently taking too long was shared by other interviewees. The example of bed nets was often mentioned in the interviews; one academic saying it took 25 years for bed nets to be distributed on a mass scale. There has to be a stronger cooperation between the different sectors for this process to go faster. This can be seen in the quote below:

“*The issue there is probably the lack of integration of the different programmes. There is probably the tendency all over the world for everyone to work in their own little niche. […] What should matter most is taking the health and the wellbeing of communities at large without specializing in one specific area, health, education, tourism, finance or agriculture*” (Programme Manager, Telephone interview, May 6, 2015).

Programme managers and academics said that both sides have unrealistic expectations from each other. In order for co-operation to exist there needs to be more understanding for the limitations each group faces. Both agreed that people working in the field do not have time or financial resources to do research and scientists had to reach out with their findings. Both sides also agreed that every sector works in their own niche; lacking a collective goal and barriers needed to be removed so that people can work together.

## Discussion

This study was started with no a priori expectations on the level of agreement or otherwise amongst participants. Disagreements were recorded in terms of the practicality and value of incorporating evolutionary principles into operational aspects of malaria control, but recorded a consensus that the principle is important for research and preventing resurgence. The reasons for this tension were partly uncovered in the responses given—seemingly there is a lack of understanding on both sides of the constraints impinged on the other side. The problem in many cases appears not to be a lack of stakeholder knowledge of the role of other actors, but the fact that potentially effective strategies towards more sustainable control cannot be implemented into programmes due to lack of funding, lack of human resources, poor infrastructure, lack of political will, poor collaboration between different sectors and poverty.

Participants agreed that resistance is a problem that arises because pathogens, vectors and humans co-evolve; and that it is the role of scientists to study this relationship and to develop and recommend new control methods. Interviewees collectively acknowledged that evolutionary principles have not been incorporated into current control efforts, and all participants agreed that control programmes cannot and should not be examining evolutionary principles. However, participants also agreed that evolutionary principles should not be disregarded from control programmes.

The results of the study are based on a convenience sample of practitioners and should be interpreted in that context. As a scoping exercise the aim was to test whether discussions on the subject would elicit meaningfully differential responses. Nonetheless, from these results it can be initially inferred that some form of EM could have a role to play in promoting sustainable control of malaria. The basic premise of EM is that clinicians and other stakeholders are trained in principles of evolutionary biology so that when faced with a health problem requiring a solution, they can reach into their personal knowledge base and use evolutionary principles to help inform their answer. Principles of EM can be used to interpret operational results [14] and also act as a connection point between evolutionary theory and applied public health strategies to make these more effective and sustainable [14].

Elements of evolutionary theory have been previously applied in a several medical-research domains including assessing the post-trial selection pressure potential of HIV vaccines [15], testing theories underpinning the aetiology of hypertension [16] and understanding how historic climate change may have selected for specific alleles involved in metabolic disorders [17].

In terms of malaria, basic-science research projects have identified a number of genetic factors corresponding to acquired immunity [18]. Wider consideration of the evolutionary underpinnings of virulence, including negative selection [19] have led to the suggestion that an evidence-based resistance-management strategy taking the absolute fitness of the parasite would ensure interventions are evolution-proof. This approach complements a suggestion by Read and colleagues [8] to target old, infected mosquitoes, so as to make resistance redundant in terms of the reproductive success of the vector.

What is clear from these and other examples is that the inclusion of evolutionary theory into medically important domains of enquiry can give clearer insights at the level of research. For effective management of medical disorders like those above, as well as any other disease that has developed as product of evolutionary processes, there are potential gains from transferring not just the knowledge gained from these studies but also a more basic understanding of the rationale and theory that underpins the investigation. This is where the basic framework of EM could assist.

One reason why EM has so far not been incorporated is possibly due to the process by which research results are translated into practice. At the level of basic research, caveats are discussed and made reasonably prominent in the publications. But as results are translated into implementation research, those same caveats are often discussed less until they may all but disappear, to be replaced by targets once roll-out of a particular solution is undertaken by implementation organizations. The basic research continues, but as this study shows, it is often difficult for stakeholders, involved with programme management who may be recruited only at the implementation stage, to understand the science in the way it is presented. Even if they do understand the science, they may not be in a position to apply that knowledge due to the limitations of their role.

At the point of roll-out, budgets are set which may or may not be sufficient to deal with all contingencies. In this study, programme managers cited lack of funding within control budgets as the biggest challenge to sustainable control of malaria. Cost, and particularly cost-effectiveness, is clearly a major factor in determining the choice of a particular intervention. But even for established interventions such as LLINs and indoor residual spraying, estimating the cost-effectiveness is far from straightforward due to a combination of factors related to such issues as local endemicity, climate, levels of immunity, transactional costs, [20]. Long-term benefits of more expensive components of sustainable control such as monitoring and surveillance are also rarely considered [21]. This lack of wider considerations is perhaps the reason why DDT, despite growing evidence of resistance, is still widely used as it is the cheapest insecticide [22].

Given that selection pressures are constantly acting on vectors and parasites, it seems sensible to suggest that any assessment of the cost-effectiveness of an intervention should ideally incorporate scenarios that consider how a pathogen or its vector may adapt to selection pressures imposed by the intervention. It may emerge during scenario planning that what appears to be a cost-effective approach in the short term is less effective on a ‘whole life-cycle’ basis. In this context, whole life-cycle cost corresponds to the time and cost of elimination of malaria from the point where basic research on a particular solution is started.

The so-called ‘arms race’ is a prominent evolutionary meme in drug-and insecticide development, but the anticipation of a problem does not in itself appear to be an agent for change. This point is made evident by the lack of attention given to any other potential solutions, beyond those in the current tool box [23], in the WHO malaria strategy 2016–30 [24] Multi-sectoral collaborations between national malaria control programmes and researchers do occur and are undoubtedly helpful, but to what extent they are equipped to give agency to alternative strategies based on planning for evolutionary adaptation is not always clear.

One issue that is being given agency in a collaboration at the research-control nexus is that of better housing. The potential for better housing to reduce malaria transmission is entirely missing from the WHO malaria 2016–30 strategy, despite being considered over 20 years ago in evolutionary terms. Ewald [25] argued improved housing conditions would place a selection pressure on the pathogen to become less virulent. Additionally, by not allowing the vector to come in contact with immobile and severely sick hosts, only mild strains would be transmitted [25]. A recent systematic review [26] confirmed that better housing reduces malaria risk, and also provided the evidence for a randomized controlled trial that involved a collaboration between researchers and a national control programme, in the Gambia [27].

Ewald [25] acknowledged that building houses is more costly than distributing bed nets whilst suggesting it is more efficient in reducing transmission. Had this concept been tested contemporaneously with its generation, the malaria control community may have headed down a different implementation route, or considered housing earlier in the intervention time line.

## Conclusion

The results of this scoping project suggest that the current tension between theory and practice, revealed by participants, may be contributing to a lack of mitigation strategies against drug and insecticide resistance—issues which can ultimately cause programmes to fail [2, 28]. Parasite, vector and host are under constant selection pressure that they put on each other [12, 13]. Widespread knowledge of this phenomenon is a key part of including EM at the research-control nexus. The results of the interviews indicate that whilst the idea of EM is not generally objected, the lack of effective monitoring and collaboration between the different sectors and the lack of political will from local governments make it currently difficult to incorporate.

Participants agreed that the role of scientists was not only to carry out evolutionary research, but also make it accessible and applicable to programme managers. One suggestion from this study is that programme managers would benefit from earlier exposure to the research agenda, and training in evolutionary theory at an appropriate level. Determining the ‘appropriate level’ will require work in itself. The main recommendation from this project is, therefore, that co-ordinating organizations, including WHO and the Centers for Disease Control and Prevention, alongside scientists and programme managers, investigate how to incorporate EM at the research-control nexus. This may be achieved through applying methods of co-production [29]—a process that goes beyond knowledge transfer to bring together academics and all other stakeholders earlier on in the research process. It will be important during this process to not lose sight of the caveats, as EM is not a panacea.

## Additional files

10.1186/s12936-016-1446-8 Consent form and information sheet—interview with audiotaping.
10.1186/s12936-016-1446-8 Interview questions on evolutionary aspects of malaria and the implications for control programmes.

## Abbreviations

ACT: artimisinin-based combination therapy
DDT: dichlorodiphenyltrichloroethane
EM: evolutionary medicine
LLIN: long-lasting insecticidal net
RBM: roll back malaria
WHO: World Health Organization
