# Supplementary material for: The application of evolutionary medicine principles for sustainable malaria control: a scoping study
Source: Malar J. 2016 Jul 22;15:383. doi: 10.1186/s12936-016-1446-8 (PMC4957922; doi:10.1186/s12936-016-1446-8)
Supplement: Supplementary file 1 — 10.1186/s12936-016-1446-8 Consent form and information sheet—interview with audiotaping. [file 12936_2016_1446_MOESM1_ESM.docx]

**Attachment**

*CONSENT FORM AND INFORMATION SHEET – INTERVIEW WITH AUDIOTAPING*

**Consent to Participate in Dissertation Research**

***Principals of evolutionary medicine in programs against malaria***

**Introduction and Purpose**

My name is Denise Ocampo. I am a postgraduate student on the Evolutionary Medicine course in the Dept of Anthropology at the University of Durham, working with my faculty advisor, Dr Mark Booth from the School of Medicine, Pharmacy and Health. As part of my studies I am undertaking a short research project. I would like to invite you to take part in my research study, which investigates how principals of evolutionary medicine may be important for malaria control programs. The working hypothesis is that increased awareness of evolutionary principles might help predict changes in both parasite and pathogen natural history and give warnings about future disease scenarios.

**Procedures**

If you agree to participate in my research, I will conduct an interview with you at a time and location of your choice, preferably over Skype. The interview will involve questions about: your understanding of the background to specific malaria intervention and prevention programs, your experience in the field of parasite or vector control, your scientific perspectives on the importance of evolutionary medicine parasite, vector and host relationship, other relevant infectious diseases, your views on how malaria is perceived by the public, scientific community and government, etc.*.* It should last from 30 to 90 minutes. With your permission, I will audiotape and take notes during the interview. The recording is to accurately record the information you provide, and will be used for transcription purposes only. If you choose not to be audiotaped, I will take notes instead. If you agree to being audiotaped but feel uncomfortable at any time during the interview, I can turn off the recorder at your request. Or if you don't wish to continue, you can stop the interview at any time.

**Please enter your initials into the boxes next to each of the following statements if you agree**

| **Benefits** | **initials** |
| --- | --- |
| You agree that there is no direct benefit to you from taking part in this study. It is hoped that the research will show that incorporating the aspects of immunity, parasite-host evolution and climate change into medical management will give better prognosis and be more effective in not only saving human lives but also increasing their quality of life. |  |
| **Risks/Discomforts** |  |
| You agree that you are free to decline to answer any questions you don't wish to, or to stop the interview at any time. As with all research, there is a chance that confidentiality could be compromised; however, we are taking precautions to minimize this risk. |  |
| **Confidentiality** |  |
| You agree that your study data will be handled as confidentially as possible. If results of this study are published or presented, individual names and other personally identifiable information will not be used unless you give explicit permission for this below. |  |
| You agree that, to minimize the risks to confidentiality, we will keep your identity anonymous. |  |
| **Data retention and further use** |  |
| You agree to the PI and myself retaining the data securely for current and future analysis |  |
| **Compensation** |  |
| You agree that you will not be paid for taking part in this study. |  |
| **Rights** |  |
| ***Participation in research is completely voluntary***. You agree that you are free to decline to take part in the project. You can decline to answer any questions and are free to stop taking part in the project at any time. Whether or not you choose to participate in the research and whether or not you choose to answer a question or continue participating in the project, there will be no penalty to you or loss of benefits to which you are otherwise entitled. |  |

**Questions**

If you have any questions about this research, please feel free to contact me. I can be reached at [denise.ocampo@durham.ac.uk](mailto:denise.ocampo@durham.ac.uk).

If you have concerns/questions about the research you would like to discuss with someone else at the University, please contact: [mark.booth@durham.ac.uk](mailto:mark.booth@durham.ac.uk)

************************************************************

# CONSENT

You will be given a copy of this consent form to keep for your own records.

If you wish to participate in this study, please sign and date below.

_____________________________

Participant's Name *(please print)*

_____________________________ _______________

Participant's Signature Date

If you agree to allow your name or other identifying information to be included in all final reports, publications, and/or presentations resulting from this research, please sign and date below.

_____________________________ _______________

Participant's Signature Date
