# Supplementary material for: The application of evolutionary medicine principles for sustainable malaria control: a scoping study
Source: Malar J. 2016 Jul 22;15:383. doi: 10.1186/s12936-016-1446-8 (PMC4957922; doi:10.1186/s12936-016-1446-8)
Supplement: Supplementary file 2 — 10.1186/s12936-016-1446-8 Interview questions on evolutionary aspects of malaria and the implications for control programmes. [file 12936_2016_1446_MOESM2_ESM.docx]

**Appendix**

Interview Questions on Evolutionary Aspects of Malaria and the Implications for Control Programmes

1. Could you briefly outline the roles and responsibilities in your job?
2. What do you think is the single biggest challenge regarding eradicating malaria from the world?
3. What do you think are the biggest successes and the biggest failures of the ongoing malaria intervention programmes?
4. In your experience what are the problems that occur during the development of an intervention/control programme in regards to the 5 pillars of strategy from the WHO: management strategy, monitoring, knowledge, new tools and enabling mechanisms?
5. How do you think (if at all) that climate change is going to affect the global malaria burden?
6. Do you know of current preventative measures that are taking different climate scenarios into account?
7. What do you think is the solution to the drug resistance problem?
8. It has been argued that evolutionary management on the long run will be more effective than developing new drugs, what do you think of that statement?
9. If there is no solution what measures need to be taken in order to slow down the development of drug resistance?
10. How can this be achieved and who would be the major actors?
11. What is the main issue with malaria control in your eyes?
12. Despite global efforts it has not been possible to eradicate malaria; do you think elimination of a parasite is possible? Do you think there should be a stronger focus on control or another aspect? Do you think this would make donors doubtful and question whether to further invest in malaria programs?
13. What do you think is a possible solution to insecticide resistance?
14. What do you think of alternatives such as bio pesticides?
15. How much are they incorporated at the moment?
16. Control programmes such as Roll Back Malaria are aiming for an overall vector control coverage, if insecticide resistance is not a failure but an unavoidable outcome as a response to these man-made selection pressures what measurements need to be taken?
17. What do you think about insecticide resistance arising through agriculture?
18. How important do you think knowledge about parasite-vector-host interaction is for you field in malaria and for intervention programmes in general?
19. What is known about trade-offs between virulence and transmission?
20. Studies have been done that indicate that reducing transmission by vector control may not reduce the global malaria burden on the long run. What do you think of this?
21. The competition between the malaria strains in a host increases virulence. By limiting the harm that is done by a parasite in a host rather than preventing the infection in the first place also increases virulence. Do you know if these factors are taken into account by programmes?
22. How much evolutionary knowledge is currently incorporated in your area of the field?
23. Do you think evolutionary aspects should be in cooperated (more) into the control programmes and that possible foresights could help in the discussed issues? Why/Why not?
24. What do you think about a future vaccine? Do you think there are hidden dangerous for example, adding an additional selective pressure to the parasite?
25. Immunisation does not stop the within host competition and selects for more virulent strains within a host, what implications do you think this will have for malaria elimination or eradication?
26. Symptoms of malaria may be more severe in immune-suppressed hosts (i.e. AIDS) are these cases given special attention?
27. Do you think the public needs to be made aware of certain malaria issues?
28. Do you think one of these issues is evolutionary knowledge?
29. Malaria has many grave impacts on society, which one do you think can and needs to be addressed first in addition to fighting the disease?
30. What problems do you see arising in 50 years’ time?
31. In what areas and fields do you think more work needs to be done?
32. Would you like to add any last comments?
